# La calle como espacio de producción de cuidados: el proyecto terapéutico singular y el manejo de la tuberculosis en personas en situación de calle durante la pandemia de covid-19 en la ciudad de Río de Janeiro

**DOI:** 10.18294/sc.2024.4774

**Published:** 2024-03-06

**Authors:** Aline Azevedo Vidal, Karla Santa Cruz Coelho, Emerson Elias Merhy

**Affiliations:** 1 Magíster en Atención Primaria de Salud. Gerente en Servicios de Salud, Secretaría Municipal de Saúde, Prefeitura do Rio de Janeiro, Rio de Janeiro, Brasil. alineavidal86@gmail.com Prefeitura do Rio de Janeiro Secretaría Municipal de Saúde Prefeitura do Rio de Janeiro Rio de Janeiro Brazil alineavidal86@gmail.com; 2 Doctora en Salud Colectiva. Profesora asociada. Instituto de Ciências Médicas, Centro Multidisciplinar. Universidade Federal do Rio de Janeiro, Campus Macaé, Rio de Janeiro, Brasil. karlasantacruzcoelho@gmail.com Universidade Federal do Rio de Janeiro Instituto de Ciências Médicas Centro Multidisciplinar Universidade Federal do Rio de Janeiro Campus Macaé Rio de Janeiro Brazil karlasantacruzcoelho@gmail.com; 3 Doctor en Salud Colectiva. Profesor titular, Instituto de Ciências Médicas, Centro Multidisciplinar, Universidade Federal do Rio de Janeiro, Campus Macaé, Rio de Janeiro, Brasil. emerhy@gmail.com Universidade Federal do Rio de Janeiro Instituto de Ciências Médicas, Centro Multidisciplinar Universidade Federal do Rio de Janeiro Campus Macaé Rio de Janeiro Brazil emerhy@gmail.com

**Keywords:** Personas sin Hogar, Grupo de Atención de la Salud, COVID-19, Tuberculosis, Brasil, Homeless People, Healthcare Team, COVID-19, Tuberculosis, Brazil

## Abstract

Desde la perspectiva teórica de la cartografía de la micropolítica del trabajo vivo en acto, el objetivo fue analizar el proceso de trabajo del equipo del “consultorio en la calle” con sede en una unidad de atención básica de la ciudad de Río de Janeiro, Brasil, en el manejo de casos de tuberculosis, en el contexto de la pandemia de covid-19. Se trata de una investigación exploratoria con enfoque cualitativo. Entre mayo y diciembre de 2021, se entrevistaron a siete profesionales del equipo consultorio en la calle, y se realizó observación participante con registros en diario de campo. De las entrevistas surgieron tres ejes temáticos relacionados con la población en situación de calle en el contexto de la pandemia covid-19: 1) Desafíos, potencialidades y fragilidades del cuidado de la tuberculosis; 2) Construcción de redes de cuidados intersectoriales para el seguimiento de las personas con tuberculosis; y 3) La calle como espacio de producción de cuidado: el proceso de trabajo del consultorio en la calle en el manejo de la tuberculosis. Se concluye que la atención a personas en situación de calle con tuberculosis en el contexto de la pandemia covid-19 requiere no solo de la gestión de protocolos clínicos, sino también de la construcción de un trabajo compartido con la red intra e intersectorial. Además de la tarea de estar en el territorio, el servicio ambulatorio del territorio también debe ser un servicio ambulatorio de la calle, especialmente en lo que respecta al tratamiento de la tuberculosis.

## Abstract (en)

From the theoretical perspective of the cartography of the micropolitics of living labor in action, the objective was to analyze the work process of the “street clinic” team based in a primary care unit in the city of Rio de Janeiro, Brazil, in the management of tuberculosis cases in the context of the Covid-19 pandemic. This is an exploratory qualitative research. Between May and December 2021, seven professionals from the street clinic team were interviewed, and participant observation was conducted with field diary records. Three thematic axes emerged from the interviews related to people experiencing homelessness in the context of the Covid -19 pandemic: 1) Challenges, potentialities, and weaknesses of tuberculosis care; 2) Building intersectoral care networks for monitoring individuals with tuberculosis; and 3) The street as a space for care production: the work process of the street clinic in tuberculosis management. It is concluded that caring for people experiencing homelessness with tuberculosis in the context of the Covid -19 pandemic requires not only managing clinical protocols but also building shared work with the intra and intersectoral network. In addition to the task of being in the territory, the outpatient service in the territory must also be a street outpatient service, especially regarding tuberculosis treatment.

## INTRODUCCIÓN

La tuberculosis es una enfermedad transmisible que continúa siendo un problema de salud pública relevante a nivel mundial^1^^,^^2^. En el caso de Brasil, enfrentar la tuberculosis persiste como un gran desafío para la salud pública. Los avances logrados en los años previos a la pandemia de covid-19 se estancaron o se revirtieron^1^ y Brasil se encuentra entre los 20 países con el mayor número estimado de casos de tuberculosis y de coinfección tuberculosis-VIH^2^.

Al estratificar el municipio de Río de Janeiro por Áreas de Planificación en Salud, se observa una alta incidencia en todas las áreas en comparación con la incidencia nacional (32,0/100 mil hab.) y estatal (67,4/100 mil hab.), lo que está lejos de las metas acordadas por la OMS^3^. Tal como se señala el *Boletín Epidemiológico* del Municipio de Rio de Janeiro, “en los últimos 10 años, la proporción de interrupciones en el tratamiento de la tuberculosis en el municipio permanece por encima de la meta preconizada por el Ministerio de Salud de hasta el 5%, con una reducción entre 2013 y 2015 y un aumento significativo a partir de 2016”, alcanzando el 16,7% en 2020. En 2021, se observó una disminución en este porcentaje de interrupción (14,3%)^3^.

Entre 2015 y 2022, se ha observado un aumento de casos nuevos de tuberculosis entre personas consideradas más vulnerables a la enfermedad, como la población en situación de calle, que presentó un bajo porcentaje de cura, con una disminución de este porcentaje a lo largo de los años, que pasó del 37,6% en 2019 al 33,9% en 2021. Además, en este período se observó un aumento en la proporción de muertes entre los casos notificados en población en situación de calle. Esta población presenta especificidades y complejidades para la implementación de acciones de prevención, diagnóstico y tratamiento de la tuberculosis^3^.

Según el *Censo de Población en Situación de Calle en Río de Janeiro 2022*^4^, al comparar el número de personas en situación de calle en 2022 (7.865 personas) respecto del Censo de 2020 (7.272 personas), se observa un aumento del 8,15%. Sin embargo, al observar solo a las personas que dormían efectivamente en las calles, el aumento fue del 15%. En este subconjunto, hubo un aumento del 3,11% para aquellas personas que estaban en escenas de uso de drogas y del 18,39% para aquellas que solo estaban en las calles. El total de personas consideradas en situación de calle, alojadas en instituciones, también aumentó un 12,02% en comparación con 2020^4^.

El consultorio en la calle ha sido una oportunidad de inclusión y garantía de derechos en 2022 para la población en situación de calle. Esta modalidad de equipo, establecida en el marco de la Política Nacional de Atención Básica de 2011, y reglamentada por las Resoluciones 122 del 25/01/2011 y 123 del 25/01/2012, tiene como objetivo atender a las personas en situación de calle desde la perspectiva integral de la atención médica^5^.

El equipo del consultorio en la calle aborda los problemas clínicos más comunes entre las personas en situación de calle, siendo la enfermedad de tuberculosis la más prevalente debido a las condiciones sociales y de salud derivadas de deficiencias nutricionales, consumo de alcohol y otras drogas, privación del sueño, falta de seguridad, infección por VIH-sida, edad avanzada y falta de cuidado de la salud que afectan la función inmunológica y aumentan la probabilidad de desarrollar tuberculosis^6^.

Algunas poblaciones son consideradas prioritarias para el control de la enfermedad, debido al mayor riesgo de contraer tuberculosis, como profesionales de la salud, personas que viven con VIH-sida, personas privadas de libertad, individuos en situación de calle, pueblos indígenas y contactos de tuberculosis resistente^7^. De esta manera, el “Plan Nacional para el Fin de la Tuberculosis como Problema de Salud Pública: Estrategias para 2021-2025” incorporó como pilar de prevención y atención integral centrada en la persona con tuberculosis, un objetivo específico dirigido a las poblaciones más vulnerables a la tuberculosis, con el propósito de intensificar y aumentar la visibilidad de las acciones estratégicas para calificar la atención a estas poblaciones^8^.

Además de los desafíos en el manejo y enfrentamiento de la tuberculosis por parte del equipo del consultorio en la calle, la Organización Mundial de la Salud declaró, en marzo de 2020, el estado de pandemia de covid-19. Ante este escenario, hemos sido testigos y experimentado un movimiento de reorganización de los servicios de salud en tiempo real para abordar esta demanda tan intensa como heterogénea. Se han establecido nuevos protocolos clínicos, se han creado nuevos flujos de trabajo, se han fortalecido ciertas modalidades de atención y estructuras para la asistencia sanitaria, además de la resignificación de funciones de diferentes espacios^9^.

Frente a este escenario en el que se indicaban diariamente medidas de prevención, cuidado y combate al covid-19 para toda la población brasileña, y ante las condiciones de vulnerabilidad experimentadas por la población en situación de calle, atravesadas por cuestiones psicosociales generadoras de sufrimientos físicos y emocionales, se recomendó que los equipos del consultorio en la calle, formados por profesionales de diversas disciplinas, que brindaban atención integral a la salud de esta población de manera itinerante, fomentaran el desarrollo de acciones compartidas e integradas con las Unidades de Atención Básica, ampliando la construcción de nuevas formas de actuación frente a las urgencias generadas por la pandemia^10^.

### Micropolítica del trabajo vivo en acto como producción de cuidado en salud

Desde la perspectiva de la micropolítica del trabajo vivo en acto, el proceso de trabajo del equipo del consultorio en la calle se caracteriza por el uso de tecnologías leves, como el acogimiento, el vínculo, el cuidado como forma de comunicarse con el sujeto para la producción de un proyecto terapéutico^11^. La estrategia utilizada por el consultorio en la calle con personas con tuberculosis debe contar con creatividad, horizontalidad e interdisciplinariedad, destacando el diálogo que permite que las personas en situación de calle compartan sus experiencias, sentimientos, vivencias y opiniones, buscando el reconocimiento de las potencialidades y limitaciones o fragilidades de las acciones frente a la tuberculosis.

La práctica del consultorio en la calle, con su apuesta por la proximidad y la actuación en el territorio y desde sus singularidades, muestra que el proceso de trabajo se da a través de subdivisiones, a partir de planes de intervención del equipo de trabajo. A su vez, estos planes requieren la gestión de procesos de trabajo más específicos^12^. La complejidad del trabajo de los equipos del consultorio en la calle sugiere la adopción de modelos centrados en una visión ampliada del cuidado, considerando las singularidades de las personas, sus necesidades y contextos de vida, lo que implicaría la construcción de proyectos terapéuticos singulares, compartidos y flexibles^13^.

La calle, como espacio de producción de cuidado, es un lugar en el que la necesidad imperiosa del compartir se expresa de manera intensiva. Es necesario reconocer al otro, sus argumentos, sus deseos, de manera tal que los equipos del consultorio en la calle puedan contribuir desde la reducción de daños, como una posibilidad de aproximación. De esta manera, la reducción de daños, entendida como una ampliación de la potencia de los existires diversos también es una modalidad de proyecto terapéutico compartido junto a las personas en situación de calle como herramienta para enfrentar la tuberculosis, ampliando la posibilidad de cuidados que tengan sentido para los usuarios^14^.

La producción o el carácter productivo del cuidado se piensa desde la micropolítica del trabajo vivo en salud, entendiendo el cuidado (en salud) como un “acontecimiento productivo intercesor”^15^, es decir, reconoce que en el encuentro profesional-persona usuaria, el cuidado se expresa como un acontecimiento caracterizado por acciones humanas que, en última instancia, todas son reconocidas como producción desde la perspectiva micropolítica: “las prácticas de salud, como toda actividad humana, son actos productivos, ya que modifican algo y producen algo nuevo”^16^.

En este sentido, al pasar del paradigma de la biomedicina al del cuidado centrado en la micropolítica del trabajo vivo en acto, se habilitan las tecnologías relacionales, que son centrales en el tratamiento de la tuberculosis. El éxito para enfrentar la tuberculosis aumentará cuando se incorporen tecnológicas en el proceso de cuidado de la tuberculosis, que equilibren los arreglos tecnológicos del cuidado hacia las tecnologías leves. El seguimiento con medicamentos y tratamientos es importante, pero para que impacten en los resultados del tratamiento de la tuberculosis, es necesario generar nuevas experiencias de acogida y de valorización del saber del otro sobre su propia existencia^17^.

El equipo del consultorio en la calle, en tanto dispositivo de salud capaz de contribuir a cambios en el modelo de atención de esta población, con la construcción de prácticas centradas en la persona usuaria, tiene el potencial para incorporar nuevas prácticas singulares de cuidado, al comprender el lugar que ocupa el territorio en la vida de estas personas. También propone que los profesionales se vean a sí mismos en el acto de cuidar y puedan situarse en procesos analíticos sobre sus formas de enfrentar la situación que viven, en el campo de la tuberculosis, adquiriendo cierto “manejo” de situaciones más complejas como el contexto de la pandemia de covid-19, en las que el conocimiento se construye a partir de la reflexión y la reformulación de la propia práctica^18^.

Desde esta perspectiva, el ámbito de las tecnologías de cuidado se convierte en un lugar privilegiado para que la y los profesionales se vean a sí mismos en el propio acto de cuidar y puedan, de esta manera, participar en procesos analíticos sobre sus formas de abordar la situación en la que viven las personas en situación de calle con tuberculosis, para abrir problematizaciones formativas y participativas que ponen los proyectos terapéuticos y sus modos de constituirse en el centro para la construcción de las prácticas de salud de los equipos del consultorio en la calle.

De esta manera, el objetivo del presente estudio fue analizar el proceso de trabajo del equipo del consultorio en la calle con sede en una unidad de atención básica del barrio de Realengo, en el municipio de Río de Janeiro, en el manejo de los casos de tuberculosis en personas en situación de calle, en el contexto de la pandemia de covid-19.

## METODOLOGÍA

Este es un estudio de naturaleza cualitativa que utiliza el método cartográfico para analizar el proceso de trabajo del equipo del consultorio en la calle con el objetivo de problematizar las prácticas de cuidado en el manejo de la tuberculosis, operacionalizadas para la población en situación de calle en el contexto de la pandemia de covid-19. El estudio permitió comprender la práctica de las y los profesionales a partir de la obtención de elementos relacionados con el cuidado, haciéndolo factible a través de la inmersión y la observación participante en la práctica de los sujetos del estudio, obtenidos a través del diario de campo.

La investigación cartográfica implica movimientos a través de varios “mundos” o las conexiones entre ellos, inventando mundos de atención médica en cada momento en que se produce un nuevo campo de investigación^19^. Esta metodología no recopila datos, sino que los produce en acto. Se considera relevante porque se esfuerza en acceder al plano relacional de la micropolítica del trabajo vivo en salud, de modo que el cuerpo de la persona que investiga afecta y se deja afectar por el campo de investigación, en una interferencia mutua, ya que la producción intensiva de encuentros y afectos fluye libremente entre el investigador y el campo, un proceso que constituye el propio campo de investigación o la “parte” principal de él en este enfoque, aquella parte en la cual la persona que cartografía se esfuerza más en sumergirse.

Según Slomp Junior *et al*.^19^, la estrategia cartográfica se centra en la producción de registros empíricos del trabajo de campo de cada investigación, inicialmente relacionada con el formato de “diario de campo”. A lo largo de las investigaciones, la producción de memoria registrada en los diarios de campo resultó ser una fuente fundamental para realizar análisis procesales de los hallazgos empíricos. Se percibió que, independientemente de las peculiaridades de cada situación de investigación, no se trataba de un diario de campo “convencional”, sino que había en esto una forma de registrar los procesos cartográficos de constitución de planos discursivos bastante peculiares.

### Campo de investigación: escenario y sujetos de investigación

El municipio de Río de Janeiro cuenta con siete equipos vinculados a la Atención Básica en Salud, que trabajan directamente con la población en situación de calle, que constituyen los equipos del consultorio en la calle. La ciudad es bastante heterogénea, presenta diferentes grados de desarrollo y, por lo tanto, desigualdades en la distribución y utilización de los recursos disponibles, incluidos los servicios de salud. El equipo del consultorio en la calle opera en los siguientes barrios del Área de Planificación en Salud 5.1: Deodoro, Vila Militar, Campos dos Afonsos, Jardim Sulacap, Magalhães Bastos, Realengo, Padre Miguel, Bangu, Gericinó y Senador Camará.

El estudio se realizó en una Unidad de Atención Básica del barrio de Realengo, con sede en la Clínica de la Familia Faim Pedro del Área de Planificación en Salud 5.1. Esta Unidad de Atención Básica opera con la población en situación de calle y cuenta con un equipo del consultorio en la calle. La elección de este equipo se debió a que era el campo de actuación profesional de la primera autora de este trabajo.

Los sujetos del estudio fueron los profesionales del equipo del consultorio en la calle: tres agentes sociales, una enfermera, una terapeuta ocupacional, una trabajadora social y una persona que brinda soporte al consultorio en la calle. Se llevó a cabo una reunión para describir la investigación, en la unidad de base del equipo con la investigadora y los profesionales. Todas las personas integrantes del equipo se ofrecieron a participar en la investigación y consintieron voluntariamente en colaborar con el estudio, dieron su consentimiento para participar en este estudio después de la presentación, explicación, entrega y firma del formulario de consentimiento informado y voluntario por parte de los participantes y la investigadora.

Los criterios de inclusión fueron: profesionales que formaban parte del equipo en el momento del trabajo de campo y que trabajaban con la población en situación de calle, durante al menos 12 meses. Los criterios de exclusión fueron: profesionales que estaban ausentes de sus prácticas con la población en situación de calle, ya sea por vacaciones, licencia médica o ausencia por pertenecer al grupo de riesgo de covid-19.

La recopilación de datos se llevó a cabo entre mayo y diciembre de 2021, mediante la aplicación de un guion de entrevista semiestructurada, junto con la observación participante y la construcción de un diario de campo. La entrevista se desarrolló utilizando un guion semiestructurado, con preguntas abiertas que permitían que las personas entrevistadas expresaran libremente sus pensamientos sobre el tema.

Las entrevistas fueron audiograbadas, transcritas íntegramente, organizadas y, después de la lectura, se extrajeron y agruparon los fragmentos que estaban directamente relacionadas con el objeto del estudio, especialmente aquellos que contenían temas recurrentes, de manera de elaborar una lectura comprensiva y crítica, respaldada por los registros del diario de campo de la investigadora. Para facilitar el análisis de las transcripciones, a cada entrevistado se le asignó un código seguido de un número. Para garantizar el anonimato, los nombres mencionados fueron reemplazados por iniciales. Se utilizó la técnica de análisis de contenido, adaptada para cumplir con la propuesta del presente estudio. Se llevó a cabo una exploración previa de las respuestas de la entrevista, y a través de la lectura flotante se seleccionaron temas que revelaran posibles categorías.

La observación participante se realizó durante la inmersión en el escenario de la investigación, acompañando al equipo del consultorio en la calle en todo momento. En el proceso de observación, la investigadora utilizó una guía que contenía los elementos a observar y anotar en el diario de campo. Las anotaciones se realizaron inmediatamente después de las observaciones e impresiones de la investigadora, con el objetivo de reducir la pérdida de información relevante. Dado que el diario de campo alude a las especificidades de las prácticas, en la primera página de cada conjunto de anotaciones se incluyó un encabezado con título, fecha, hora, lugar, actividades/situaciones experimentadas (identificación), nombre del observador y número del proceso de organización de prácticas de cuidado en el territorio, realizada por el equipo del consultorio en la calle.

Finalmente, se procedió al agrupamiento de temas. El método analítico consistió en dar visibilidad a las relaciones que constituyen una realidad dada, en la cual la investigadora también está implicada. El análisis, a la luz de la cartografía, posibilita movimientos por varios “mundos”, a través de las conexiones entre ellos, y conduce a una redefinición de las fronteras entre subjetividad y objetividad^19^. El análisis cartográfico se alinea con otras aproximaciones de investigación e intervención, y asume diferentes procedimientos que permiten el análisis de la implicación en la investigación y, por ende, el análisis de la participación. Como efecto del análisis, se produce un reposicionamiento del lugar de los participantes en la investigación. El análisis cartográfico permite, a lo largo de toda la realización de la investigación, el acceso a una objetividad que, en lugar de fijar un sentido unívoco, tiende a multiplicar sentidos. La cartografía afirma tal paradoja a través de una actitud analítica como agente de singularización^20^.

De esta manera, se establecieron los siguientes ejes temáticos para la presentación y discusión de los resultados relacionados con la población en situación de calle en el contexto de la pandemia covid-19: 1) Desafíos, potencialidades y fragilidades del cuidado de la tuberculosis; 2) Construcción de redes de cuidados intersectoriales para el seguimiento de las personas con tuberculosis; y 3) La calle como espacio de producción de cuidado: el proceso de trabajo del consultorio en la calle en el manejo de la tuberculosis. 

El estudio fue sometido al Comité de Ética e Investigación y a la Comisión Nacional de Ética en Investigación, a través de la Plataforma Brasil, cumpliendo con las Resoluciones 466/2012 y 510/2016, que regulan las investigaciones con seres humanos, y la Norma Operativa 001/2013, bajo el protocolo CAAE: 40957020.90000.5699.

## RESULTADOS

### Desafíos, potencialidades y fragilidades del cuidado de la tuberculosis

*¡El covid-19 llega a Brasil! La (in)visibilidad de las personas en situación de calle... El enfrentamiento de las vulnerabilidades cotidianas... El creciente desempleo... El aumento de la pobreza... Las prácticas de salud reducidas... El debilitamiento del SUS... ¿A dónde llevará todo esto?* (Diario de Campo, 2021)

Según la Organización Mundial de la Salud^2^, el impacto negativo en los indicadores de tuberculosis puede explicarse, en parte, por las altas demandas de salud generadas por la pandemia en los servicios de salud. Esto se debe a que, en muchos países, los recursos destinados a la tuberculosis (humanos, financieros y otros) se reasignaron a dar respuesta al covid-19, lo que llevó a interrupciones en los servicios de salud y a más retrocesos en el control de la tuberculosis.

Las personas entrevistadas llaman la atención sobre cómo la pandemia de covid-19 ha tenido impactos directos en las actividades de los servicios de salud, tanto en las consultas ambulatorias de rutina como en las actividades en el territorio, y en los servicios y actividades sociales y de promoción de la salud que quedaron suspendidos debido a las medidas de aislamiento, siendo uno de los desafíos destacados en el manejo de la tuberculosis en personas en situación de calle.

*Articulación con la red. Las unidades se enfocaron mucho en el covid-19, hubo un cambio en el funcionamiento de las unidades y eso dificultó bastante.* (D10)

En ese contexto, la pandemia de covid-19 ha sido un desafío exponencial para toda la Atención Básica, especialmente para los equipos de consultorio en la calle, dada la necesidad de reorganizar el servicio para combatir la enfermedad, además de mantener las actividades de rutina que deben garantizarse, a pesar de la reconfiguración e incorporación de nuevos procedimientos. Hacer frente a la pandemia requiere una adecuada protección de las y los profesionales de la salud, ofreciendo entornos de trabajo seguros, evitando que se conviertan en medios de transmisión. Para ello, deben realizarse cambios en la organización del proceso de trabajo de acuerdo con la realidad local, promoviendo la educación permanente con y entre los equipos multidisciplinarios, y el potencial para identificar el riesgo de enfermedad y garantizar la atención oportuna^21^.

Otra dimensión es comprender que cuidar del territorio no es solo intervenir en el cuadro clínico: es circular en los territorios con la debida protección y con una agenda de trabajo para el período de la pandemia, generada con diferentes actores locales. Así, es posible construir una capacidad operativa desde otra modalidad de cuidado de la pandemia, a través de una amplia red de cuidados de proximidad con profesionales capacitados, evitando el abandono de quienes tuvieran síntomas y de sus contactos, actuando con mayor consistencia en el control de la transmisión comunitaria y construyendo otras posibilidades para el distanciamiento social. Es necesario construir redes de cuidado que se desplieguen en una multiplicidad de tecnologías de cuidado basadas en los territorios de la vida y el trabajo de las personas, utilizando herramientas mucho más manejables desde el punto de vista del costo, pero sobre todo con la capacidad de intervenir de manera efectiva en la contención del contagio comunitario, asegurando una oferta suficiente de pruebas, equipos de protección personal, información, apoyo articulado con capacidad de acoger, dialogar y compartir acciones^9^.

*Uno de los desafíos es fortalecer la red intersectorial* [...]. *La comprensión de la tuberculosis es algo que algunos dispositivos de atención social desconocen, como el seguimiento del tratamiento. El equipo del consultorio en la calle realiza esta intersección no solo con dispositivos de salud sino también con los de asistencia. Sensibilizar a los profesionales que no conocen el manejo de la tuberculosis en la población en situación de calle, debido a que es una población estigmatizada y, con la tuberculosis, la situación empeora aún más. Potenciar el seguimiento de una manera desestigmatizada por parte de las instituciones.* (K12)

Otro aspecto fue que el cronograma de actividades del equipo se reorganizó, teniendo en cuenta la necesidad de equilibrar las acciones en los albergues, en las calles, en las unidades del territorio del área programática y en la unidad base del equipo. El punto de fragilidad fue cuando, en el momento más crítico de la pandemia, cuando la población necesitaba más cuidados, las y los profesionales del equipo comenzaron a enfermarse. Hubo una reducción abrupta del personal debido a la contaminación por covid-19, y otra parte quedó fuera debido a la resolución que definía los grupos de riesgo, disminuyendo la efectividad en las intervenciones.

*La fragilidad fue que perdimos profesionales. Los profesionales tuvieron que ausentarse por enfermedad, comorbilidad. Había momentos en que algún profesional estaba enfermo de covid-19. Un equipo grande se redujo y realmente fue un poco complicado realizar todas esas intervenciones.* (L11)

Honorato y Oliveira^22^ refuerzan la idea de que el equipo de salud debe acudir a satisfacer las necesidades y no dejar que se presenten como demanda. Además de los cuidados *in situ* en el territorio durante las intervenciones, hubo un aumento en la demanda en la Unidad de Atención Básica por parte de la población en situación de calle. Ante el temor a contagiarse, solicitaban documentación para recibir ayuda del gobierno, manifestaban diversas cuestiones de salud, incluso de pacientes que ya no se encontraban en nuestra área de cobertura. Las solicitudes eran variadas: hablaban sobre la dificultad para conseguir alimentos e incluso sobre los “*garimpos*” para obtener algo de dinero, ya que su red de apoyo, como algunos establecimientos comerciales e instituciones religiosas que entregaban comida, tenían sus actividades reducidas y/o cerradas debido a la cuarentena decretada como resultado de la pandemia de covid-19.

*El problema es que los demás servicios también tenían miedo de ir a la calle debido al covid-19, no solo el servicio formal sino también el informal, donde la población en situación de calle tiene más acceso a alimentos y ropa, todos estaban cerrados* [...]. *Fue uno de los desafíos en términos de alimentación para que tomen la medicación, sin comer, medicación fuerte, tomar la medicación con hambre.* (R12)

Cuando se les preguntó acerca del potencial del equipo en el manejo de la tuberculosis en la población en situación de calle en el escenario de la pandemia de covid-19, las respuestas de las personas entrevistadas muestran la importancia del trabajo compartido, el vínculo y las estrategias de abordaje en el territorio. El equipo del consultorio de la calle lleva a cabo acciones *in situ* para la población en situación de calle, de manera itinerante y compartida con otros puntos de atención médica, siendo un dispositivo de la red que hace de puente entre la calle y los servicios, y también conecta esa red de atención para calificar y garantizar la atención de las necesidades de estas personas^23^. Entre las acciones desarrolladas durante la pandemia de covid-19, el equipo del consultorio de la calle retomó el “Café Cultural”, que se consolidó como una estrategia importante de reducción de daños y construcción de vínculos entre las y los profesionales y las personas en situación de calle. Es una estrategia de cuidado y fortalecimiento de vínculos con la población que vive en áreas marcadas por diversas vulnerabilidades, donde la interacción cotidiana en la localidad desde un servicio de baja exigencia ha facilitado el acceso de estas personas usuarias a la red asistencial y ha facilitado la detección y el seguimiento exitoso de diversos problemas complejos, como la tuberculosis.

*La fragilidad en el contexto de la tuberculosis en tiempos de pandemia fue principalmente la falta de profesionales, teníamos que ocuparnos de todo, la falta de la camioneta, el desconocimiento de la enfermedad y algunos usuarios se fueron de los lugares donde estaban buscando comida porque todos los comercios estaban cerrados. La fortaleza está en nuestro trabajo con la tuberculosis y en el trabajo compartido. Compartimos con las otras clínicas e incluso cerca de donde se encuentra el usuario.* (R11)

El equipo no encuentra dificultades para acceder a los lugares de mayor concentración de población en situación de calle, ya que la vinculación con los usuarios que ya están en tratamiento para la tuberculosis facilita la identificación y el acceso a los demás. Así, el consultorio en la calle utiliza como principal herramienta la relación personal, ya que esta posibilita una aproximación cuidadosa a la persona usuaria, adaptando las acciones a desarrollar a partir del establecimiento de vínculos de confianza, en busca de una interlocución posible.

En este sentido, uno de los desafíos del trabajo del consultorio en la calle se refiere a la integridad de las personas en situación de calle afectadas por la extrema desigualdad, que se ve aún más empobrecida por las precarias condiciones de vida. Hay un conjunto de factores que afectan la salud física y mental, que corresponden a los modos de vida en aglomerados, la inadecuación alimentaria, del sueño, del descanso y la higiene, la susceptibilidad a enfermarse y al uso y abuso de sustancias psicoactivas^24^. Brindar soporte a las necesidades humanas básicas destacadas aquí, como alimentación, refugio y oportunidades para descansar y dormir, junto con la preservación de la dignidad y los derechos universales, son actitudes esenciales que posibilitan y acercan el diálogo.

### Construcción de redes de cuidados intersectoriales para el seguimiento de las personas con tuberculosis

*…cada lugar con sus desafíos... desarticulación de la red de apoyo... la crisis llega a las calles... una realidad aún más difícil!!!* (Diario de Campo, 2021)

Además, es esencial considerar que, en el contexto de la pandemia de covid-19, la inversión en el cuidado de las personas con tuberculosis y sin hogar debe realizarse de manera compartida a nivel local, en el territorio, avanzando en la articulación y fortalecimiento de las redes intersectoriales. El vínculo con las personas en situación de calle debe tener en cuenta la proximidad geográfica con la unidad básica de salud, no solo con el equipo del consultorio en la calle. El cuidado compartido y la participación en la red son imprescindibles dada la complejidad de esta población.

Al abordar la existencia de una red jerarquizada, articulada y organizada para compartir el cuidado, en un intento de garantizar la continuidad del cuidado, la integralidad y la resolutividad de la atención, uno de los objetivos del equipo es ampliar el acceso a los diferentes puntos de atención médica y la red intersectorial, integrando y articulando las acciones con los diferentes servicios, siendo una entrada articuladora y cuidadora para el Sistema Único de Salud (SUS), además de dar visibilidad a las demandas de esta población. De este modo, se plantea la necesidad de que el sector salud establezca colaboraciones con otros sectores, lo que indica que la producción de intersectorialidad será determinante para el grado de resolutividad del sistema de salud^25^.

*Nosotros tenemos en el área la asistencia representada por el CREAS* [Centro de Referência Especializada de Assistência Social]*, instituciones civiles y la sociedad civil de forma organizada.* (K1)

Otro elemento importante que surgió del trabajo de campo destaca la importancia del alineamiento de acciones y el diálogo entre los dispositivos del territorio para lograr un cuidado compartido y fortalecer la red intra e intersetorial. Es necesario preservar los espacios de intercambio de experiencias, como reuniones, colegiados y supervisión de casos, con la participación de la red de personas, equipos y servicios, respetando la singularidad de cada persona en situación de calle de manera individualizada. En cuanto a la articulación con las unidades básicas de salud para el seguimiento, es imperativo invertir en la formación de los equipos para brindar apoyo al trabajo del consultorio en la calle, permitiendo que el cuidado se construya localmente.

*La infraestructura que tenemos son las unidades básicas de salud y las clínicas de familia. Otro dispositivo es el CREAS* [Centro de Referência Especializada de Assistência Social]*, a veces también los refugios formales e informales, y ahora también contamos con el personal de seguridad*. (L1)

Una parte esencial y uno de los objetivos del trabajo de los equipos del consultorio en la calle es activar los territorios existenciales en los encuentros que se producen diariamente entre el equipo y las personas usuarias, saliendo de la lógica de la unidad básica de salud y encontrándose con la vida y su complejidad en las calles, especialmente con el trabajo de los agentes sociales^26^. La interlocución con los dispositivos de asistencia social está presente en el manejo de la tuberculosis en la población en situación de calle, así como en las unidades básicas de salud del Área Programática 5.1. Sin embargo, la red del equipo no debe estar restringida a una región, ya que los usuarios provienen también de otros lugares, siendo una de las atribuciones de los profesionales de los equipos del consultorio en la calle ayudar en la reinserción en una nueva red social y de atención a la salud en un nuevo territorio.

*La unidad de salud o clínica de la familia, con el agente comunitario presente en el territorio. Otro dispositivo social que generalmente trabaja con nosotros en el mismo territorio es el CREAS* [Centro de Referência Especializada de Assistência Social]*, brindando atención a las personas en los mismos lugares que visita el equipo del consultorio en la calle*. (L2)

La relación intersectorial entre la Secretaría Municipal de Asistencia y Desarrollo Social y la Secretaría Municipal de Salud ha evolucionado mucho, ya que actualmente existe disposición de las gestiones para este entendimiento, y los problemas sociales siempre son discutidos en las reuniones de territorio^27^. De esta manera, el equipo del consultorio en la calle debe intentar unir la red, siendo responsable de articular todos los puntos de atención necesarios para el recorrido de la persona en situación de calle en la red de atención integral, tratando de resolver las dificultades y trabajar con el tema.

Es importante destacar que el trabajo del equipo del consultorio en la calle, en la búsqueda de establecer articulaciones intersectoriales en el contexto de la pandemia de covid-19, estuvo presente de manera más intensa en varios momentos, especialmente en los pacientes con seguimiento por tuberculosis que requerían hospitalización o regularidad asistencial. Dada la importancia de las acciones intersectoriales, la articulación con otros dispositivos de salud es imprescindible para la integralidad de la atención y la resolución de la atención, pero al mismo tiempo genera tensiones, dificultades y barreras en el proceso de trabajo de los equipos del consultorio en la calle, al no considerar criterios como la gravedad, la situación de vulnerabilidad de la persona en situación de calle y la oportunidad para aquella persona usuaria que opera en la lógica del “aquí y ahora”, es decir, en la noción de que “el tiempo es el aquí y ahora”, refleja la inmediatez de las respuestas de los equipos y las demandas de las personas usuarios^28^.

Al recapitular los puntos comunes entre las personas entrevistadas con relación a la construcción del trabajo en red para el manejo de la tuberculosis en la población en situación de calle, queda claro que la actuación en el territorio está orientada por una propuesta intersectorial y por la identificación de las posibles redes formales e informales disponibles para que el cuidado sea integral, e involucre no solo la salud, sino también otros sectores.

*Con la pandemia de covid-19 algunas colaboraciones se hicieron* más *fr*á*giles, aunque en un contexto así se ven los espacios que son potentes. El comedor popular también era uno de los dispositivos hasta hace poco tiempo, las donaciones de las iglesias.* (K5)

En este sentido, las ofertas de cuidado de proximidad tienen una potencia para la gestión de las crisis, como posibilidad de enfrentar la pandemia en la producción micropolítica de los encuentros cotidianos, superando los límites de la subjetivación del modelo biomédico que también han marcado las ofertas de base comunitaria. El llamado “cuidado de proximidad” se ha definido como el cuidado realizado lo más cerca posible de las personas usuarias de un determinado territorio. Este concepto, en la práctica, va más allá de una noción de proximidad en términos geográficos, de delimitación física de áreas de cobertura. Se trata de dispositivos que se proponen estar, de manera activa, cerca de los problemas cotidianos de la vida comunitaria, acercándonos a una comprensión de “territorio” como un escenario de las acciones humanas^9^.

Es en la intersectorialidad donde existe la posibilidad de abordar ampliamente las cuestiones y dificultades más complejas de la población en situación de calle en sus múltiples dimensiones. A pesar de las dificultades para convertir la intra e intersectorialidad en una práctica cotidiana, esa la articulación ha sido una de las estrategias más utilizadas en el proceso de viabilización y acceso de la población en situación de calle a las políticas públicas^29^.

*Cuando el paciente necesita ser internado tanto por cuestiones sociales como por deterioro clínico, la cama disponible sale de los hospitales de referencia de tuberculose de Rio.* (L6)

Por consiguiente, es necesario actuar de manera intersectorial y colectiva, articulando con dispositivos locales para potenciar el cuidado ofrecido. Es fundamental ser sensible al espacio (territorio y redes) en donde se desarrolla la vida de quienes residen en la calle, reflexionar sobre este lugar vivo, observar cómo está articulada la red de cuidado para poder insertar las acciones en el territorio vivo. La calle es indispensable para el manejo de la tuberculosis en la población en situación de calle.

### La calle como espacio de producción de cuidado: el proceso de trabajo del consultorio en la calle en el manejo de la tuberculosis

*Abordaje y acogimiento… cuidado integral a la persona en situación de calle… transformación del plano de las quejas en circuitos de demandas… ser referencia y aproximarse al territorio de la vida…* ¡*cartograf*í*a m*óvil *de los modos de vida de la persona en situación de calle!* (Diario de campo, 2021)

La calle como espacio de cuidado debe tener en cuenta que las demandas de la población en situación de calle presentan niveles diferenciados y deben ser acogidas en espacios diferenciados. De esta manera, la calle, la red, la unidad de salud, los servicios y los espacios deben articularse para el seguimiento. La organización de la red ha sido una constante fuente de tensiones para la práctica de los profesionales del consultorio en la calle, siendo más acentuada en el contexto de la pandemia de covid-19.

*El enfoque y la construcción del vínculo los buscamos en el territorio, activamos la red informal con aquellos que están ahí dándonos apoyo para avanzar en ese tratamiento. Cuando el usuario enfrenta dificultades que lo van a llevar a interrumpir el tratamiento, solicitamos la internación social porque muchas veces él mismo señala cuando no va a poder continuar*. (L9)

Con frecuencia, durante los abordajes y discusiones de casos, la red informal o “territorios afectivos” tuvieron gran importancia en el seguimiento de las personas usuarias con tuberculosis durante la pandemia de covid-19, brindando apoyo al equipo para llevar a cabo el tratamiento con personas en situación de calle. Es necesario entender que las personas usuarias, como redes de existencia, se producen “en-mundos”, se “in-mundizan” y componen ciertos modos de realizar la producción de cuidado. Las calles son, entre tantos territorios existenciales, un lugar donde las existencias actúan y se producen como redes vivas, donde los usuarios construyen sus propias redes con diferentes conexiones^11^.

Ante la observación del trabajo de campo y a través de las similitudes en las percepciones de las personas entrevistados con respecto al proceso de trabajo, queda claro que la práctica de cuidado se sustenta en la lógica de ir a las calles a través de abordajes que establezcan un proceso continuo entre la calle y la unidad de atención, no solo para identificar o realizar la búsqueda activa de personas usuarias en tratamiento para tuberculosis, sino también para abrir posibilidades y construir un proceso de cuidado en el cual la persona en situación de calle ocupe una posición protagonista. El trabajo no es solo un conjunto de intervenciones, sino una apuesta por un seguimiento secuencial según cada circunstancia acordada en el proyecto terapéutico singular.

*Tenemos reuniones semanales y, en el caso de casos positivos, se crea o al menos se discute un plan para las dosis observadas y la rutina que vamos a seguir para estar siempre presentes con él, no solo para llevar la medicación, sino para cualquier cosa que necesite.* (K7)

El proyecto terapéutico singular que se implementa en cada caso se elabora y se discute grupalmente, buscando acciones dirigidas a la producción de cuidado y la reducción de vulnerabilidades, y como una forma de brindar mayor resolutividad al equipo. El proyecto terapéutico singular debe basarse en las historias de vida de las personas, en sus condiciones de vida, en sus necesidades y deseos. Cabe destacar que las orientaciones y la construcción de estrategias de cuidado propuestas por el proyecto terapéutico singular deben darse a través del diálogo en las consultas en la unidad básica de salud o en las calles^30^.

De esta manera, el proyecto terapéutico se produce a partir del trabajo de las personas del mundo de la salud en relación con las personas usuarias, que también actúan todo el tiempo por sí mismas. Este trabajo se centra en el trabajo vivo en acto, el cual ocurre en la acción relacional entre la persona trabajadora y la persona usuaria-ciudadana, y su atributo principal es el autogobierno de quien trabaja sobre su propio actuar en el proceso de trabajo.

Durante el abordaje se comprendió que la calle debe considerarse como una herramienta de apuesta clínica y no solo como un territorio físico, sino como el territorio de vida de esta población. El equipo del consultorio en la calle debe ser sensible a las herramientas que la calle proporciona para la producción de cuidado, partiendo de los acuerdos construidos y de estrategias de acompañamiento individualizadas para cada caso.

Las dimensiones invisibles, entendidas como las dinámicas relacionales que los usuarios o grupos establecen en un determinado territorio y los lugares que estas dinámicas ocupan en las vidas de los sujetos, deben considerarse para la organización de un proceso de trabajo más resolutivo. Entendiendo las tecnologías relacionales (construcción de vínculo) como un elemento que amplifica la resolutividad, y que contribuye a sortear las carencias estructurales del cuidado en la calle. Se observó que estar en la calle, experimentar una cercanía con las personas en situación de calle, lidiar con sus condiciones de vida, establecer empatía, escucha y prácticas enfocadas a encontrar potencialidades en sus palabras fortalece el vínculo, permitiendo construir en conjunto un plan de cuidados, que se reformule junto con la persona usuaria según posibilidades y necesidades establecidas por ellas.

*Se amplió la mirada del equipo hacia el cuidado del paciente en situación de calle con tuberculosis. La tuberculosis en medio de la pandemia nos hizo desacelerar y tener una mirada más profunda dentro de la pandemia para estos pacientes. Dado que el covid-19 vulnera mucho a la persona y aún más a una persona con tuberculosis y covid-19, el equipo protegió mucho más al paciente, incluso estando en una situación de extrema vulnerabilidad. El equipo fortaleció el vínculo y ajustó el proceso de trabajo para prestar más atención a las personas que están con tuberculosis.* (K13)

El proceso de trabajo del equipo del consultorio de la calle tiene como foco de actuación la calle y, al vincular las acciones de atención básica, trabaja en la lógica de la promoción del cuidado. Esto requiere la construcción de una red más amplia, establecida en la práctica diaria de los profesionales. El equipo visita instituciones y busca llevar a cabo acciones conjuntas, actuando de manera dinámica a través de enfoques, escucha calificada, retroalimentación y seguimiento de las personas usuarias de las instituciones involucradas. El objetivo es garantizar un cuidado responsable y efectivo, favoreciendo el cuidado compartido^31^.

Desde el punto de vista asistencial, el equipo organiza los flujos de trabajo durante su práctica diaria y realiza un seguimiento compartido con la unidad básica de salud de referencia de la persona usuaria, asumiendo la responsabilidad del cuidado y actuando como gestor del proyecto terapéutico singular. Esto implica acompañar a la persona usuaria en el tratamiento de la tuberculosis, garantizando el acceso a otros niveles de asistencia, así como la contrarreferencia para mantener el vínculo del usuario con el servicio y desarrollar el cuidado de manera longitudinal.

*La población con tuberculosis termina recibiendo un poco más de cuidado incluso moviéndose por el territorio, y a veces desapareciendo. Entendemos la dinámica del territorio donde está insertada la población en situación de calle. El equipo se fortaleció en el proceso de trabajo para evitar el abandono y para que esta persona con tuberculosis no se enferme por covid-19. El proceso está bien articulado, prácticas de cuidado y orientaciones con escucha calificada con promoción de la salud.* (K14)

Otra de las acciones del consultorio en la calle es acompañar a las personas usuarias derivadas a servicios de alta complejidad, como estrategia para ampliar la resolutividad del equipo. Esta disponibilidad es descrita en otras investigaciones^32^ como positiva, aunque surge de situaciones negativas, como la preocupación del equipo por el tratamiento que recibirá la persona en situación de calle y las dificultades de la persona usuaria para expresar sus demandas y necesidades^32^.

Según los testimonios de las personas entrevistadas, en el contexto de la pandemia de covid-19, las personas con seguimiento por tuberculosis hicieron que el proceso de trabajo del equipo del consultorio en la calle tuviera un papel fundamental en la vinculación de todo el territorio del Área de Planificación en Salud 5.1. Se buscó lograr una atención integral, con un enfoque humanizado y universal, orientado al potencial de vida, cuidado y educación para la autonomía. Se entiende que la calle tiene su propio movimiento y la población en situación de calle, ante la pandemia de covid-19, instó a los profesionales a replantear el manejo de la tuberculosis, ya que la producción del cuidado no debía limitarse a acciones protocolares.

*Siempre tratamos de hacer supervisión diaria. Hacemos un acuerdo con el paciente para determinar lo que es mejor para él. Entonces, el paciente vendrá al equipo cuando esté cerca de la unidad base, o nosotros iremos hasta él. A veces, utilizamos equipos y buscamos a alguien que pueda ayudarnos, con la red de apoyo que el equipo cuente para la asistencia en el tratamiento supervisado de tuberculosis, tanto de apoyo formal como informal. A veces, se encuentra cerca de un comercio o una iglesia.* (L4)

De acuerdo con las directrices del Equipo Pop Rua^33^^,^^34^, el proceso de trabajo de los equipo del consultorio en la calle incluye el seguimiento longitudinal, la construcción de vínculos, la promoción de la autonomía, la cartografía constante del territorio y la actuación en la calle, la creación de instrumentos e indicadores adecuados a la realidad del territorio, el establecimiento de referencias, la escucha ampliada, la construcción y la promoción de espacios de atención diversos, la presión sobre la red, y la ocupación y definición de espacios políticos en la ciudad.

El equipo entrevistado tiene una organización del proceso de trabajo, con una organización previa de todas las acciones programadas y la construcción de la agenda de actividades. La organización de las actividades se realiza semanalmente con el objetivo de establecer un cronograma de acciones específicas para los miembros del equipo, según la necesidad de cada caso, que resulta una herramienta importante para el manejo de personas en situación de calle en seguimiento por tuberculosis. La reunión de equipo se entiende como una estrategia para ampliar la resolutividad. También se menciona como un espacio de convergencia para diferentes discusiones sobre el proceso de trabajo, la sistematización de las acciones del equipo del consultorio en la calle en la rutina para la organización antes y después de las actividades en la calle, el monitoreo y la evaluación de las acciones de los equipos, además de ser un espacio de educación permanente^25^.

*Nuestra acogida al residente de la calle es la herramienta que utilizamos para fortalecer el vínculo. Con esto vamos evaluando a cada persona y recolectamos las muestras de esputo.* (M3)

Por lo tanto, es fundamental ampliar las reflexiones sobre los cuidados relacionales como una herramienta potente para el proceso de trabajo del equipo del consultorio en la calle en el manejo de la tuberculosis entre la población en situación de calle en el escenario de la pandemia de covid-19. También es importante discutir en las reuniones la forma en la que los equipos llevan a cabo el cuidado, la estructura y la organización de la red, con el objetivo de avanzar en el apoyo coordinado y la aplicabilidad de la acogida, el diálogo y el accionar compartido con los diversos dispositivos del territorio.

## DISCUSIÓN

El equipo del consultorio en la calle busca ampliar el acceso integral a la salud de las personas en situación de calle, actuando de manera itinerante y brindando atención directa a esta población en diversos espacios. Históricamente, las personas en situación de calle han estado excluidas de los servicios sociales y de salud, lo que aumenta su riesgo de enfermedad, especialmente por enfermedades infectocontagiosas como la tuberculosis.

La población en situación de calle es el grupo más susceptible para contraer tuberculosis y, por lo tanto, el trabajo del equipo del consultorio en la calle es crucial para enfrentar la enfermedad. Reconocen el papel de los determinantes sociales en el proceso de salud-enfermedad-cuidado y realizan la búsqueda activa de esta población, ofreciendo investigación, diagnóstico y tratamiento para la tuberculosis en el lugar donde viven y, además de proporcionar atención integral en salud, uno de los objetivos es garantizar que estas personas tengan acceso al Sistema Único de Salud (SUS).

El estudio de Hino *et al.*^35^ señala que la tuberculosis sigue siendo un problema de salud pública y un desafío en diversos países. El control de la enfermedad depende de la combinación de acciones intersectoriales, institucionales y sociales, además de aquellas que inciden en los factores biológicos. Los autores advierten que la falta de vivienda lleva a una reducción en el éxito del tratamiento, y recomiendan, para lograr la adhesión al tratamiento, la creación de vínculos entre profesionales de la salud y las personas que viven en situación de calle, sugiriendo la implementación de acciones como el proyecto terapéutico singular, la colaboración con la Red de Apoyo Psicosocial y la oferta de incentivos para garantizar el éxito del tratamiento.

Según Silva *et al*.^36^, en su estudio sobre diagnóstico y tratamiento de la tuberculosis en población en situación de calle, se evidenciaron elevados porcentajes de abandono del tratamiento y fallecimientos. Estos altos porcentajes reafirman las conclusiones de estudios realizados en Brasil y en países europeos sobre la condición de vivir en situación de calle como un factor que agrava el tratamiento de la tuberculosis y aumenta la ocurrencia de fallecimientos^37^^,^^38^^,^^39^.

Los resultados de este estudio concuerdan con la bibliografía en lo que respecta a los significados atribuidos al control de la tuberculosis, demostrando la importancia de la atención brindada por el equipo del consultorio en la calle para mejorar el acceso de las personas en situación de calle a los servicios de salud y servicios sociales, así como para realizar derivaciones a otros servicios. Este enfoque contribuye al diagnóstico temprano, inicio inmediato del tratamiento y seguimiento hasta la obtención de la cura^40^.

A pesar de la importancia del tema, el análisis de la producción científica destaca la necesidad de estudios que se centren no solo en la comprensión de la ocurrencia de la enfermedad en las personas en situación de calle, sino principalmente en las formas de enfrentar la tuberculosis. Dado que el equipo del consultorio en la calle actúa como articulador de la red de atención a la salud de las personas en situación de calle, es necesario dar visibilidad al tema e invertir en tecnologías de cuidado capaces de abordar una realidad compleja y permeada por aspectos biopsicosociales inherentes a la vida en la calle.

## CONSIDERACIONES FINALES

Este estudio analizó el proceso de trabajo del equipo del consultorio en la calle con sede en una unidad de atención básica del barrio de Realengo, en el municipio de Río de Janeiro, lo que permitió identificar algunas singularidades de las acciones desarrolladas para el manejo de la tuberculosis en la población en situación de calle en el contexto de la pandemia de covid-19.

Se considera relevante que la intersectorialidad sea un requisito básico para obtener mejores resultados en el manejo de la tuberculosis, especialmente en el contexto de la pandemia de covid-19. El trabajo compartido con la red, ya sea formal o informal, favorece la articulación para la realización del tratamiento, la identificación y evaluación de síntomas respiratorios, la realización de exámenes como la prueba rápida molecular, radiografías de tórax y pruebas rápidas de VIH, así como el registro clínico calificado para la gestión de casos de usuarios con tuberculosis.

El equipo del consultorio en la calle implementó estrategias educativas que buscaron promover la salud entre la población en situación de calle frente a la pandemia de covid-19, involucrándose en procesos dialógicos de intercambio de información y aclaración sobre la enfermedad y sus formas de prevención, además de realizar la articulación intersectorial. Estar en el territorio es fundamental, pero no suficiente. Además de la tarea de estar en el territorio y construirse a través de él, la clínica en el territorio debe ser también una clínica de la calle, especialmente en lo que respecta al manejo de la tuberculosis. Esto está relacionado con la capacidad de ampliar o variar los modos de cuidado, habitando y acompañando los territorios de vida de los usuarios, que operan a través del vínculo y la experiencia de estos territorios, produciendo intervenciones basadas en las experiencias vividas en ellos.

Esta investigación, a través de la reflexión derivada de las experiencias de los profesionales, visibilizó las acciones de cuidado basadas en la corresponsabilidad, el establecimiento de la escucha y la posibilidad de acuerdos diversos. Se destacó la importancia de salir de la perspectiva centrada en la enfermedad y dirigirse hacia el sujeto, centrándose en el territorio de vida (el sujeto y sus relaciones) y abordando los desafíos que el territorio presenta, construyendo el proceso de trabajo desde espacios compartidos y toma de decisiones colectivas, abriendo espacio para que la calle ingrese a la red.

En este sentido, el lugar de la persona en situación de calle con tuberculosis, con sus conocimientos y prácticas, se convierte en la base para la construcción de proyectos terapéuticos singulares como estrategias de cuidado del equipo del consultorio en la calle frente a la pandemia de covid-19.
